# Predicting transcription factor site occupancy using DNA sequence intrinsic and cell-type specific chromatin features

**DOI:** 10.1186/s12859-015-0846-z

**Published:** 2016-01-11

**Authors:** Sunil Kumar, Philipp Bucher

**Affiliations:** Swiss Institute for Experimental Cancer Research (ISREC), School of Life Sciences, EPFL, Station 15, Lausanne, CH-1015 Switzerland; Swiss Institute of Bioinformatics (SIB), EPFL, Station 15, Lausanne, CH-1015 Switzerland

## Abstract

**Background:**

Understanding the mechanisms by which transcription factors (TF) are recruited to their physiological target sites is crucial for understanding gene regulation. DNA sequence intrinsic features such as predicted binding affinity are often not very effective in predicting in vivo site occupancy and in any case could not explain cell-type specific binding events. Recent reports show that chromatin accessibility, nucleosome occupancy and specific histone post-translational modifications greatly influence TF site occupancy in vivo. In this work, we use machine-learning methods to build predictive models and assess the relative importance of different sequence-intrinsic and chromatin features in the TF-to-target-site recruitment process.

**Methods:**

Our study primarily relies on recent data published by the ENCODE consortium. Five dissimilar TFs assayed in multiple cell-types were selected as examples: CTCF, JunD, REST, GABP and USF2. We used two types of candidate target sites: (a) predicted sites obtained by scanning the whole genome with a position weight matrix, and (b) cell-type specific peak lists provided by ENCODE. Quantitative in vivo occupancy levels in different cell-types were based on ChIP-seq data for the corresponding TFs. In parallel, we computed a number of associated sequence-intrinsic and experimental features (histone modification, DNase I hypersensitivity, etc.) for each site. Machine learning algorithms were then used in a binary classification and regression framework to predict site occupancy and binding strength, for the purpose of assessing the relative importance of different contextual features.

**Results:**

We observed striking differences in the feature importance rankings between the five factors tested. PWM-scores were amongst the most important features only for CTCF and REST but of little value for JunD and USF2. Chromatin accessibility and active histone marks are potent predictors for all factors except REST. Structural DNA parameters, repressive and gene body associated histone marks are generally of little or no predictive value.

**Conclusions:**

We define a general and extensible computational framework for analyzing the importance of various DNA-intrinsic and chromatin-associated features in determining cell-type specific TF binding to target sites. The application of our methodology to ENCODE data has led to new insights on transcription regulatory processes and may serve as example for future studies encompassing even larger datasets.

**Electronic supplementary material:**

The online version of this article (doi:10.1186/s12859-015-0846-z) contains supplementary material, which is available to authorized users.

## Background

Genes are regulated by transcription factors (TF) binding to physiological target sites in the genome. TFs may bind directly to target sites through sequence-specific protein-DNA interactions, or indirectly through protein-protein interactions with other TFs [1]. Understanding the mechanisms by which TFs are recruited to their target sites is essential for the understanding of gene regulation. For a long time, research in this area has been hampered by the lack of powerful assays to study TF binding events in vivo. This has drastically changed with the advent of the ChIP-seq technology which allows for comprehensive, genome-wide mapping of all in vivo bound sites of a given TF in a particular cell type at near base-pair resolution [2]. What has become clear from ChIP-seq experiments is that the intrinsic binding specificity of a TF can only partly explain the in vivo site occupancy patterns, which in addition were found to be tissue-specific [3]. The recruitment of TFs to target sites thus depends on both DNA-intrinsic properties and cell type specific covariates.

The intrinsic DNA binding specificity of a TF is commonly represented by a so-called position weight matrix (PWM) [4]. A PWM is a *N ×* 4 matrix whose elements define the TF’s binding preferences for the four bases of the DNA alphabet along a binding site of length *N.* Base preferences may either be expressed as occurrence probabilities or as (additive) binding energies. PWMs are basic binding site models with known limitations. For instance, they cannot model nearest neighbor dependencies nor can they account for variable spacing between reverse-complementary half-sites of homodimeric TFs [5]. Nevertheless, it is generally agreed that at least some PWMs are good predictors of in vitro binding affinity of the corresponding TFs. Moreover, large collections of PWMs are available from public databases such as JASPAR [6]. More advanced modeling techniques have been proposed for describing more accurately the binding specificity of a TF [7] but corresponding factor-specific models are not yet available for more than a handful of TFs.

Other DNA sequence-intrinsic contextual features have been used to reduce false positive rates in PWM-based in vivo TF binding site (TFBS) prediction, for instance DNA structural properties [8, 9]. Double-stranded DNA possesses anisotropic flexibility, which determines its stability and rigidity, properties that potentially interfere with DNA-protein binding. These structural properties, which are broadly classified into (i) DNA conformation (A-DNA philicity and Z-DNA stability energy), (ii) flexibility (B-DNA twist, protein DNA twist, propeller twist and bending stiffness) and (iii) stability (duplex disruption and stability free energy, stacking energy and denaturation) are sequence dependent, and at least partly predictable from structural characteristics of dinucleotides as revealed by crystal structures of double-stranded oligonucleotides. Cross-species conservation is another sequence-derived feature that has been successfully exploited for distinguishing biologically functional TFBS (evolving under purifying selection) from non-functional ones [10].

Several recent studies have reported that TF binding is influenced (and thus potentially predictable) by chromatin contextual features such as DNA accessibility, nucleosome occupancy, or the presence of specific histone post-translational modifications [11]. This is expected as these modifications were known to be associated with gene regulation processes long before. However, with the availability of high resolution ChIP-seq data for many histone marks in many cell types (e.g. ENCODE collection) it has become feasible to use these features in a systematic manner to build predictive models of in vivo TF binding. Proof of concept comes from a number of recent studies where chromatin features have successfully been exploited to predict TF site occupancy with high confidence [12–14].

In the current work, we have combined multiple sequence-intrinsic parameters with experimental chromatin feature types and used machine-learning methods to assess their relative importance in the TF-to-target-site recruitment process, in the hope to gain new insights into transcription regulatory mechanisms. We used five diverse TFs as examples: (i) the insulator protein CTCF featuring 11 zinc finger domains, which has also been attributed diverse functions including transcriptional repression, genomic imprinting and tumor suppressor [15], (ii) JunD, a leucine zipper protein and member of the activator protein 1 (AP1) family, (iii) the transcriptional activator GABPA, the only obligatory multimeric TF within the Ets family [16], (iv) the transcriptional repressor REST, also known as neuron-restrictive silencer factor (NRSF), and (v) USF2 a member of the evolutionary conserved basic helix-loop-helix leucine zipper TF family. These factors were chosen on the one hand because they represent diverse classes of transcription factors, and on the other hand because they were extensively assayed by ChIP-Seq and other chromatin profiling techniques (*e.g.* DNase I hypersensitivity assays) in multiple cell types by the ENCODE consortium.

## Methods

### Dataset definition and download

Binding sites were represented by a single genomic position corresponding to the center of the binding regions. Two different types of TFBS collections were used in this study.

- *Genome-wide predicted sites*: PWMs for CTCF (MA0139.1), JunD (MA0492.1), GABPA (MA0062.2), REST (MA0138.2) and USF2 (MA0526.1) were downloaded from the JASPAR database [6]. The human genome assembly hg19 was scanned with these PWMs using in-house tool PWMScan (http://ccg.vital-it.ch/pwmtools) with a P-value threshold of 0.0001. Predicted sites with no or only marginal ChIP-seq tag counts (<3) in any of the cell lines analyzed were removed. Final numbers of sites for each factors were: CTCF (77878), JunD (65526), GABP (51926), REST (48171) and USF2 (37138). The midpoint of the motif was used as the reference position in the peak lists. (For PWMs of even-numbered length, the position immediately upstream of the midpoint was used).
- *ENCODE peak lists* were downloaded from GEO (see Additional file 1 for sample ids). The “narrow peak” format version was used wherever available else the “broad peak” format was used. In either case, we used the midpoint of the peak region as reference point.

TFBS lists were annotated with cell-type specific and sequence-derived quantitative (numerical) features. These annotations were stored in a table with rows corresponding to binding sites and columns to annotation features. Cell-type specific experimental features were calculated as follows:

- ChIP-seq and DNase-seq tag coverage: Files containing mapped sequence tags from a single experiment were taken from the Mass Genome Annotation (MGA) repository of the Eukaryotic Promoter Database EPD [17]. Most of these datasets were originally downloaded in BAM format from the UCSC genome browser database (http://genome.ucsc.edu/ENCODE/downloads.html). Others were generated locally by mapping the sequence reads downloaded from the short read archive (SRA) [18] to the human genome assembly hg19 with the aid of Bowtie [19]. For determining the tag counts, we used only the 5’ end positions of the mapped sequence reads. For non-histone ChIP-seq targets (TFs, cofactors, and PolII) these positions were shifted downstream or upstream towards the estimated fragment center by an appropriate distance (determined using cross correlation between + strand tags and – strand tags). The GEO sample accession numbers and specific shifting distances are given in Additional file 1. Multiple tags mapping exactly the same genome positions were counted only once per experiment. For transcription factors and corresponding cofactors (Rad21 for CTCF and FOSL for JunD) as well as for PolII, tags were counted in a window of ±100 bases relative to the TFBS center. A window of ±250 bases was used for DNase I hypersensitivity data. Histone modification tags were counted in windows of ±500 bases.
- Shape-based evaluation of DNase-seq (DNase I hypersensitivity data, also referred to as “digital genomic foot-printing” (DGF)) profiles. We essentially followed the probabilistic partitioning protocol used by Nair et al. [20] for ranking and refocusing CTCF sites. This method attempts to classify the input data into typical and atypical examples. Limited shifting of individual genomic regions is allowed to optimally match the aggregate profile of typical examples. Here, DNase-seq tags were extracted from a ±500 bp region around TFBS center positions and binned in 10 bp windows. Shifting was limited to *±*50 bp (11 shift states). The method returns a probability *p* that a given genomic region constitutes a typical example plus an optimal shifting distance *s*. The output variable *p* was used as quantitative shape-based DGF feature in our analyses.

Sequence-derived cell type-independent features:

- *Position weight matrix (PWM) score:* For predicted TFBSs, the score was obtained as a by-product of scanning the whole-genome with the PWM. For ENCODE peaks, the PWM score of the best match to the JASPAR matrix within ±100 bp form the peak center was used. For computing the PWM scores, the base probability matrices from JASPAR were converted into log-odds matrices using the following base composition of the human genome as background frequencies: A - 29 %, C - 21 %, G - 21 %, T - 29 %.
- *Base (oligonucleotide) compositions:* mono-, di-, tri, tetra- and penta-nucleotide frequencies were calculated for ±100 base sequences around TFBS sites. The sequences were split into upstream and downstream parts relative to the TFBS center. Oligonucleotide frequencies were then determined separately for the two parts and for the complete sequence region, and the frequencies obtained in this way were used as three separate annotation features.
- Nucleosome occupancy prediction: average nucleosome occupancy per base was calculated for sequences around TFBS using the model built by Kaplan et al. (Version 3.0) [21]. Sequences ±1000 bases long were used in order to avoid border effects. The final feature consists of the average score of ±10 bases around the TFBS center.
- *Structural features*: These were calculated with the R script “dinucl.R“ from the DnaFVP package (http://dnafvp.sourceforge.net/) . In essence, this script assigns a score taken from a table to each dinucleotide within a DNA sequence. The average value over the ±10 bp regions (20 dinculeotide positions) relative to the TFBS center was used as quantitative structural feature. The following 10 features were used: A-DNA philicity, Z-DNA stability energy, duplex disruption free energy, duplex stability free energy, stacking energy, DNA denaturation, B-DNA twist, protein DNA twist, DNA propeller twist, and bending stiffness.
- *Conservation and polymorphism:* A compressed version of the phastCons46way track of about 10 bp resolution was used as input [22, 23]. The corresponding track file (available at ftp://ccg.vital-it.ch/mga/hg19/phastcons/) presents conservation profiles in a “counts per position” format similar to the ChIP-seq data files. The average PHASTCONS score within ±50 bases was used as conservation feature. SNP frequencies are based on dbSNP132 (~30 million SNPs) [24]. All variants of type SNP (excluding indels) were intersected with the genomic coordinates of extended TFBS regions (±100 bp) using Annovar [25]. The average SNP count within ±10 bases was used as final SNP feature.

### Feature selection and model building

Data matrices consisting of features in columns and TF sites/peaks in rows were first normalized by centering columns on the mean and then scaled by dividing the centered values by the standard deviations of the corresponding columns.

Classifiers to discriminate strong from weak TF binding sites (top and bottom 20 %) were built and evaluated with the R functions *train* and *trainControl* from the *Caret* package. First, a given data table was randomly split into 90 % sites for model building and 10 % for testing. Support Vector Machines (SVM) and Random Forest (RF) were used to build a model under leave-one-group-out cross-validation (10 groups). Polynomial and radial basis function kernels were used with SVM. Area under the receiver operating curve (auROC) was computed with the R function *roc* from the *pROC* library and used as measure of accuracy. The *rfe* function from the *caret* library was used to carry out Recursive Feature Elimination (RFE) with SVM. In addition to RFE, which reduces the dimensionality of the parameter space in a way that takes into account correlations between features, an automatic selection of best performing SVM parameters (sigma was pre-estimated using *sigest* function form *kernlab* package and fixed for a range of cost C parameter which varied form 0.25 to 32) was used along with leave-one-group-out cross-validation to optimize models and reduce over-fitting.

Regression-based quantitative binding strength predictors were also built and cross validated with the R functions *train* and *trainControl*. The TF tag count coverage served as target variable. Cross-data set predictions were made with the R function *predict* and the relative importance of different features was extracted with *varImp* (all functions are part of the *caret* package). All sites from a given table were used in this case. SVM regression with radial basis function kernel was used as training method and a Pearson’s correlation coefficient (PCC) between measured and predicted TF tags was used as performance indicator.

## Results and discussions

**Fig. 1:**
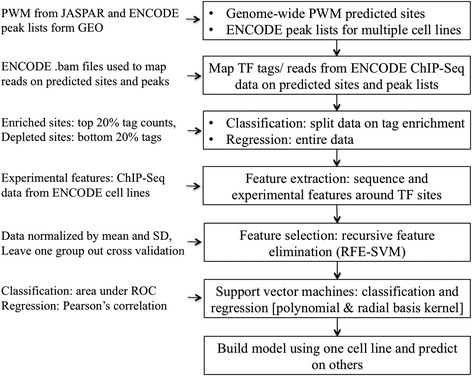
Overall workflow. Overall approach for prediction of TF site occupancy

The overall approach for prediction of TF site occupancy is outlined in Fig. 1. Two types of TFBS lists were used in this study: (i) genome wide-predicted sites resulting from a whole-genome PWM scan and (ii) cell-type specific ENCODE peak lists derived from ChIP-seq data. In both types of lists, we defined the genomic location of a TFBS by a single base position corresponding to the center of the PWM match or the peak region.

In the next step, these peak lists were annotated with the ChIP-seq tag counts from the corresponding experiments. Tags were counted in a ±100 base region relative to the TFBS center (see Methods for details). These tag counts represent binding strength estimates of an individual TF in different tissues. Note that both predicted binding sites and ENCODE peaks were retrospectively annotated with tag counts. For predicted sites, the same list was annotated with different ChIP-seq data representing different cell-types. In the case of ENCODE peak list, different lists were annotated with different ChIP-seq data pertaining to the corresponding cell types.

In two-class prediction experiments, the goal was to discriminate between strong and weak binding sites. To this end, only the top and bottom 20 % of TF binding sites (in terms of tag coverage) were used. For regression-based quantitative binding strength prediction, all TFBS of a given list were considered.

Each peak list was annotated with a large number of features that could be used as input variables for building predictors. Some of these features were directly derived from genome sequences or general annotation resources and thus invariable between different cell types. These features include a PWM-based TFBS score, base and oligo-nucleotide compositions of the binding regions (comprehensively referred to as “sequence” features, see Methods for more details), DNA structural parameters (10 in total), the distance to the nearest TSS, predicted nucleosome occupancy based on the model by Kaplan et al. [21], SNP density, and a cross-genome conservation score. Cell-type specific features included ChIP-seq derived relative abundances of 8 histone marks, PolII and co-factors known to be associated with the TFs under investigation (Rad21 for CTCF and FOSL1 for JunD). In addition, two measures of chromatin accessibility were computed from DGF. One of these measures is count-based whereas the other is shape-based (see Methods for details). The annotated peak lists were then used as data input for various machine-learning algorithms. For class prediction experiments, we used the area under the ROC curve as performance measure, for quantitative binding strength prediction, we used a Pearson correlation coefficient (PCC).

To gain preliminary insights into the relative importance and usefulness of the various features, we focused first on CTCF, a kind of model system for ChIP-seq data analysis. Specifically, we built binary classifiers to distinguish strong from weak binding sites using different features and feature classes. Classifiers were built for both predicted TFBS and ENCODE peaks. Data for the K562 cell line were used in this computational experiment.

**Fig. 2:**
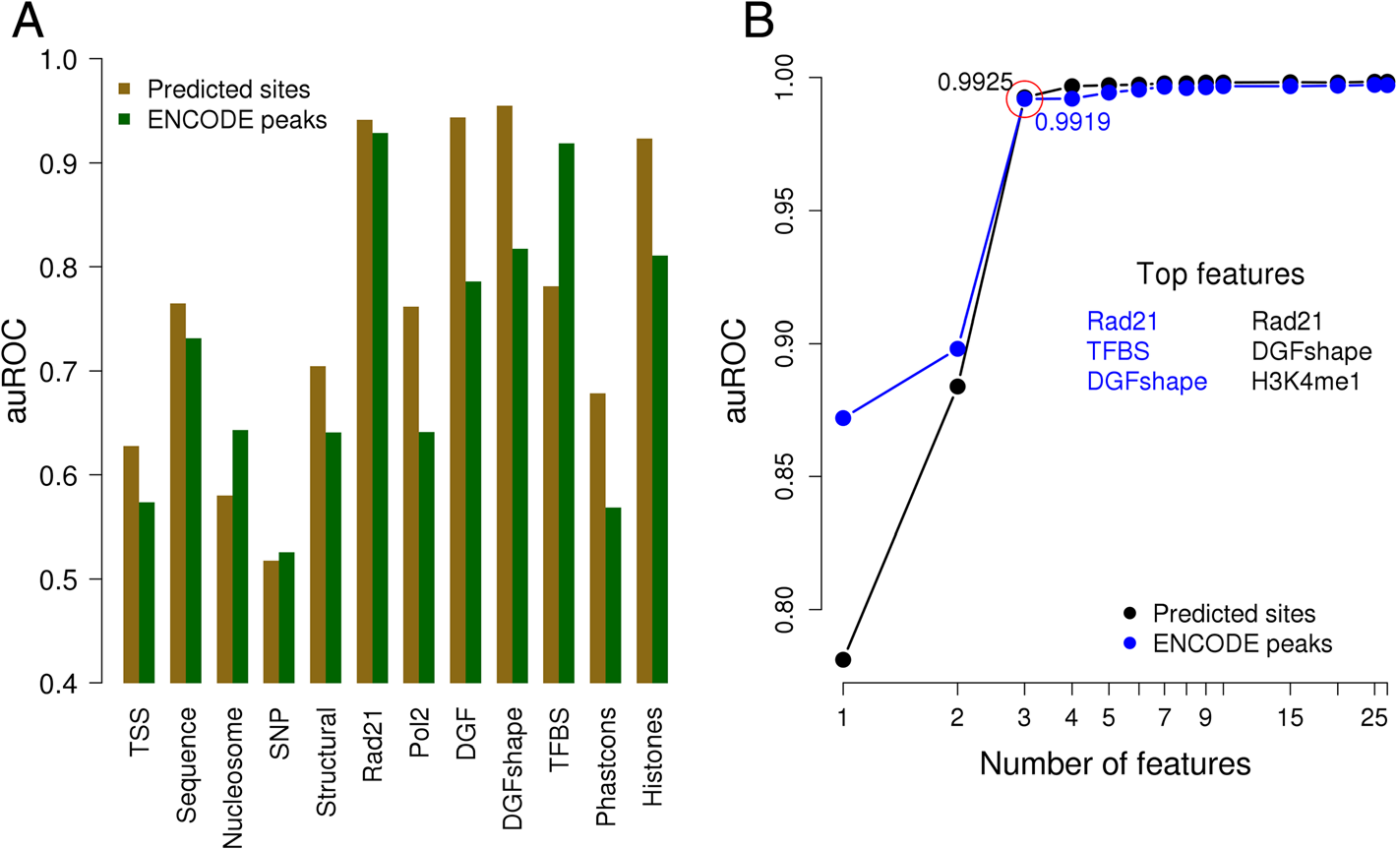
Classification results in genome wide predicted sites and ENCODE peak lists. The performance in classifying strong versus weak binding sites is reported as area under ROC curve. **a** Performance of individual feature or feature classes on CTCF sites (predicted sites and ENCODE peak lists) in K562 cell-line. **b** Feature importance assessed by recursive feature elimination (RFE-SVM)

The performance of the trained classifiers was evaluated in a 10-fold cross-validation setting. We tested three different machine-learning methods, RF and SVM with polynomial and radial basis function kernel (see Additional file 2 for a performance comparison of the three algorithms on a subset of data). The results obtained with the best performing method (SVM with radial kernel) are shown in Fig. 2a. Overall, a high auROC was achieved in binary classification for both datasets. The CTCF-interacting protein Rad21 was the pest performing feature, followed by chromatin accessibility (DGF) and the PWM-based binding score (TFBS). Histone marks together also performed quite well but may be partly redundant (correlated) with DGF. The good performance of Rad21 confirms previous reports that the majority of in vivo occupied CTCF sites in the genome are actually bound by the complete cohesin complex [26, 27].

Interestingly, experimental features such as DGF and histone mark perform better for predicted sites whereas the TFBS score is more useful for discriminating strong from weak binding sites in the ENCODE peak lists. The weaker performance of TFBS on predicted site could be explained by assuming that some of the high-scoring PWM matches reside in closed chromatin regions and thus are simply inaccessible to CTCF. Such “false positives” would be difficult to identify without experimental data, as chromatin accessibility varies substantially between cell types. Moreover, the diversity in TFBS is reduced compared to experimentally determined in vivo binding sites, as the predicted sites are selected for PWM matches with a high score. For analogous reasons, the weaker performance of DGF and histone marks on ENCODE peak lists is not surprising. In simplified terms, in vivo binding sites are sampled from accessible and active chromatin regions, resulting in reduced variability of these features as compared to predicted sites.

The predictive power of the numerous sequence features combined (over 4000 in total, see Methods) was equal or lower than the TFBS score alone. Furthermore, using that many sequence features was computationally expensive and time taking. Therefore we replaced these features in subsequent analyses by a simple G + C content feature, which we knew from parallel studies, was positively correlated with CTCF binding. Since count and shape-based DGF measures showed very similar performance, we kept only the simpler count-based measure. All other features were kept even though some showed very modest performance. The particularly weak performance of SNPs may be explained by the generally low SNP counts (often 0) in TFBS regions.

We carried out RFE, to identify the most informative individual features and to analyze the degree of redundancy between features. RFE works by backward elimination, starting with all the features and eliminating one at a time. At each step, the feature judged to be least useful for prediction is eliminated and the overall performance of the predictor is re-evaluated by cross-validation. The results of recursive feature elimination are shown in Fig. 2b. We note that for both predicted TFBS and ENCODE peaks maximal performance (0.99 accuracy) is reached with as few as three features. The cofactor Rad21 and DGF (shape-based) are part of the top features in both cases, complemented by the PWM score for ENCODE peaks and histone modification H3K4me1 for predicted sites.

In the next experiment, we used a quantitative (regression-based) prediction approach to address the following questions: (i) predict the binding strength of TFs (ii) do the previous observations concerning CTCF generalize to other TFs? (iii) Can a predictor trained on one cell type be applied to another cell type? We thus extended our study to another transcription factor, JunD, and to another cell type, H1hESC, an embryonic stem cell derived cell line. We divided the complete feature collections into two major groups: (i) chromatin-related (cell-type specific) features and (ii) sequence-derived (cell-type independent) features. Predictors were trained on each subset individually and on all the features together. As we found again that SVM with radial kernel performed better than the other two methods tested, we used this method here and in all subsequent analyses described in this paper.

**Fig. 3:**
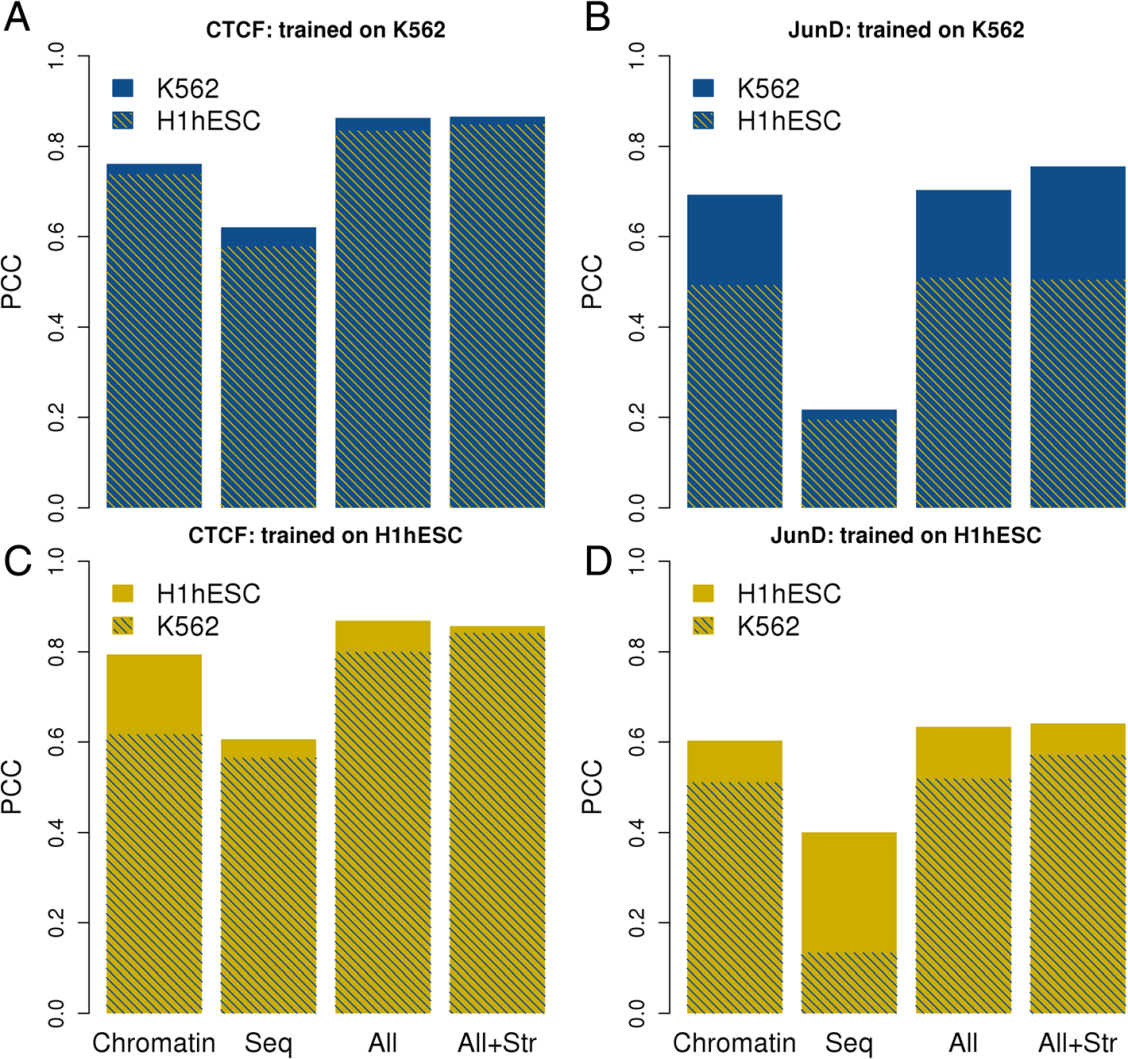
Regression results with cross cell line prediction. The bar plots reflect the prediction ability (Pearson’s correlation coefficient) of regression models trained on one cell line and tested on the same (cross-validation) or another cell line using four different feature sets on ENCODE peak lists. “Seq” consisted of sequence and annotation features; “Chromatin” features included DGF, various histone marks, PolII and co-factors (Rad21 for CTCF and FOSL1 for JunD); “All” included both of them, “All + Str” included structural features in addition to other features. Models for CTCF (**a**, **c**) and JunD (**b**, **d**) were alternatively trained on data from K562 (**a**, **b**) or H1hESC (**c**, **d**) cells

Figure 3 shows the regression results for predictions within a cell line (10 fold cross-validation) and across a different cell line. Prediction accuracy of TF occupancy levels was higher for CTCF (*R* = 0.85 to 0.91) than for JunD (*R* = 0.72 to 0.82). The lower predictability of JunD binding strength is primarily due to the poor performance of the sequence and annotation-derived features (*R* ~ 0.2). We further noticed a higher cell type specificity of the trained predictors for JunD as compared to CTCF. The model built on the K562 CTCF peak list was a relatively good predictor of H1-hESC CTCF occupancy and vice versa. However, for JunD the models performed significantly better when tested on the same cell (drop of *R* from about 0.7 to 0.5). We also explored the role of structural features in regression analysis; even though we observed a slight improvement in cross-cell line prediction, the results obtained with structural features were not a significant improvement over those obtained with sequence plus annotation features alone.

**Fig. 4:**
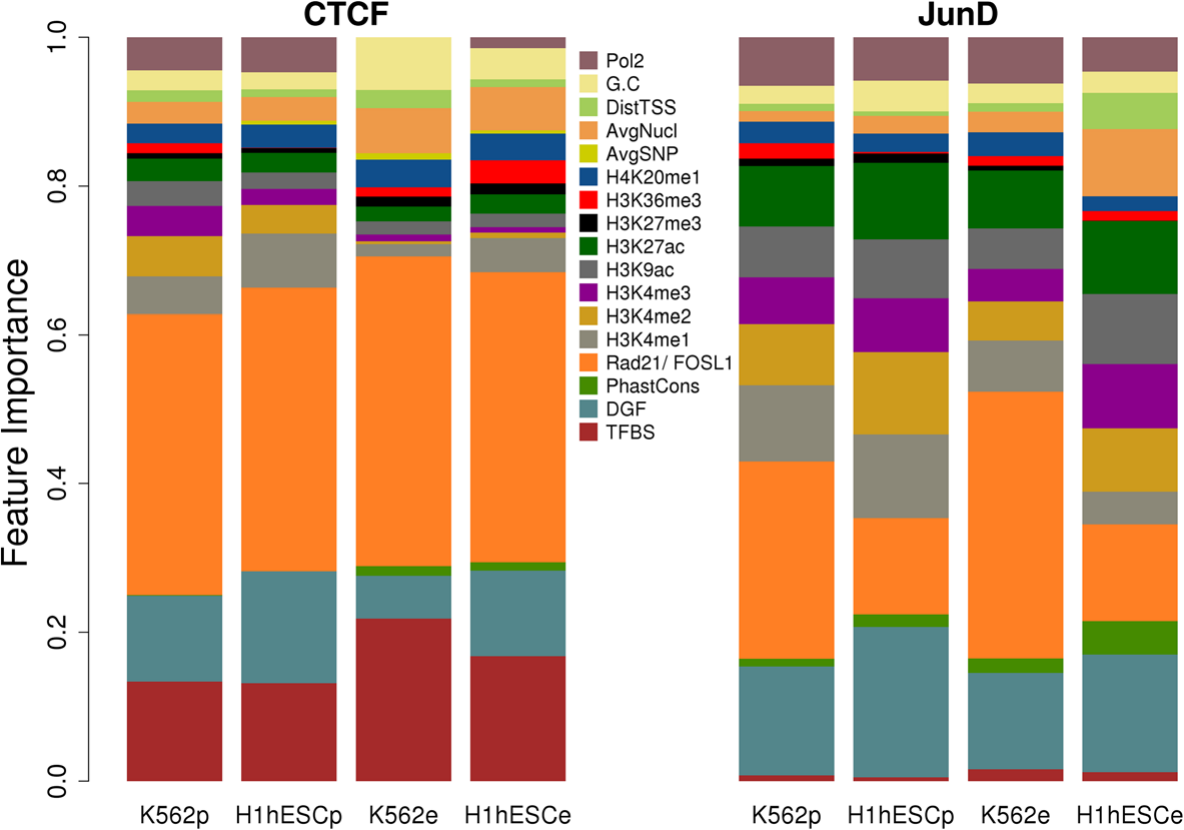
Feature importance in regression. The size of the colored areas reflect the relative importance of different features in regression models built for CTCF and JunD in different data sets. “p” and “e” in the x-axis denotes predicted sites and ENCODE peaks respectively

The machine learning method used (from the R package caret, see Methods) automatically computes “importance values” for each feature. In simplified terms, these values correspond to the reduction in prediction accuracy resulting from removal of the features. Feature importance of the binding strength predictors for predicted sites and ENCODE peaks for K562 and H1hESC cells are shown in Fig. 4. For CTCF, the importance of Rad21, DGF and TFBS together remains very high for all TFBS lists, with the relative contribution of TFBS and DGF score changing between predicted sites and ENCODE peaks as observed in the binary classification assays (Fig. 2). Other features were assigned relatively low importance and vary considerably across the four data sets. Again the trends observed with binary classification are reproduced by regression analysis. Histone marks, especially H3K4me2/3, are of comparatively higher value for predicted TFBS than for ENCODE peaks.

A markedly different picture emerges for JunD. We first note the almost total insignificance of the PWM-based binding score, which largely explains the previously observed global ineffectiveness of sequence-derived features in JunD binding strength prediction. Interestingly, this holds for predicted as well as for ChIP-seq defined binding sites. The intrinsically low binding specificity of JunD reflected by the low information content of the corresponding JASPAR PWM may partly explain this observation. We also tested a secondary PWM for JunD from the Jaspar database (MA0491.2) and observed a marginally better (but still poor) performance. In biological terms, this probably means that sequence-specific protein-DNA interactions play only a minor role among the molecular processes that recruit JunD to its physiological target sites. Another interesting observation is that the cofactor FOSL1 is assigned lower feature importance in predictors built from H1hESC cells as compared to K562 cells. A hypothetical explanation for this unexpected difference could be that JunD preferentially associates with members of the AP1 family other than FOSL1 in H1hESC cell. Generally, active histone marks are of much greater importance for predicting JunD binding strength than CTCF binding strength. Repressive histone marks (H3K27me3) and gene-body marks (H3k36me3 and H4K20me1) are of little or no use for binding strength predictions, independently of cell type.

**Fig. 5:**
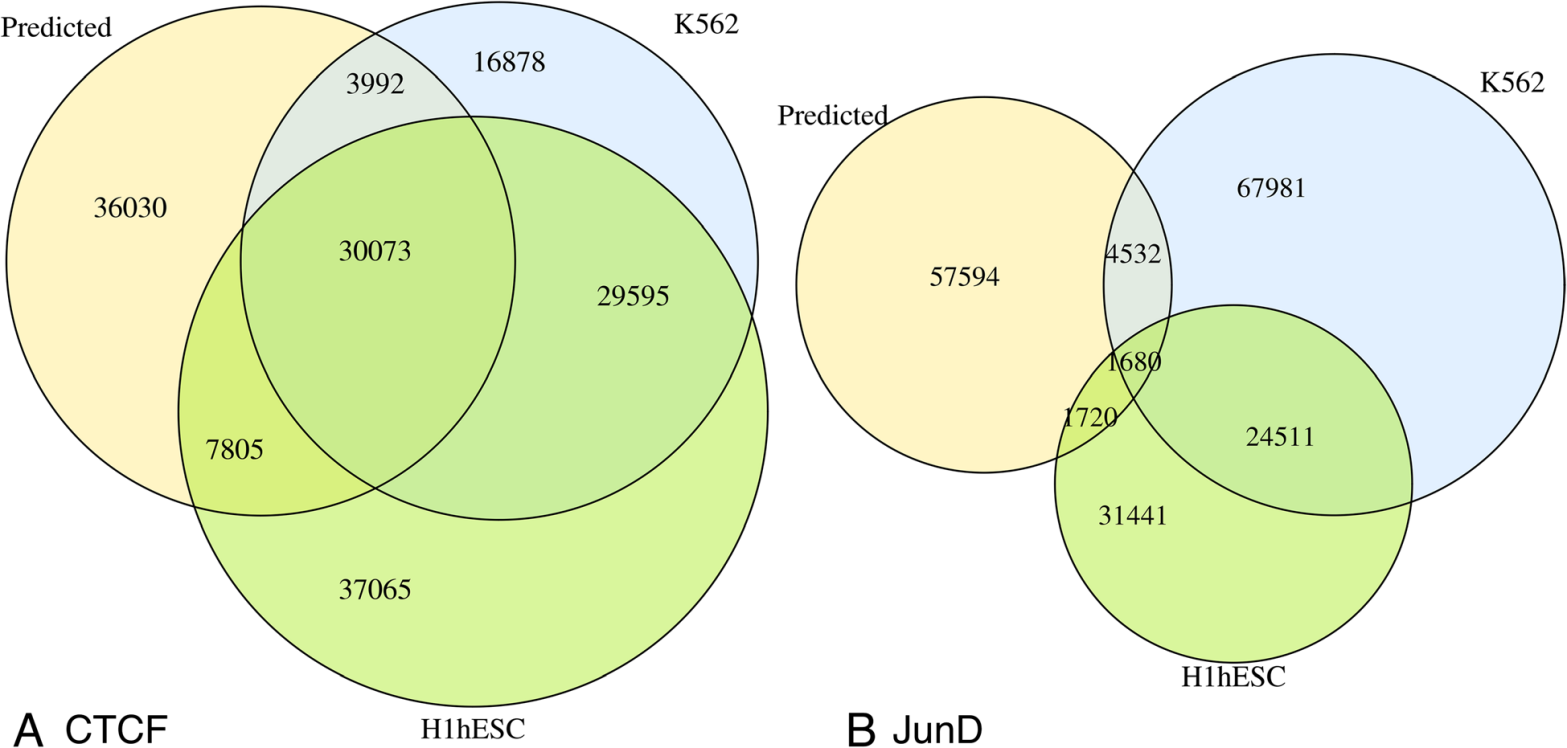
Overlap between predicted sites and ENCODE peaks. The Venn diagrams show the overlap between predicted TFBS lists and ENCODE peak lists for two cell types (K562 and H1hESC) for (**a**) CTCF and (**b**) JunD

The results presented so far suggest that the intrinsic DNA binding specificity plays a much more important role for in vivo target site selection by CTCF as compared to JunD. At the same time, the selection process appears to be much more cell type-specific in the case of JunD. These two observations would imply a smaller overlap among the TFBS lists for JunD than for CTCF. To verify that this is indeed the case, we generated Venn diagrams for the three TFBS lists (predicted sites, ENCODE peaks for K562 and H1hESC) for the two factors (Fig. 5). Indeed, there is a much smaller overlap between the TFBS lists for JunD. There are only 1,680 JunD sites (0.9 %) common to all three lists from a combined total of 189,459 sites. In contrast, there are 30,073 common CTCF sites (18.6 %) out of 161,438 sites in total.

**Fig. 6:**
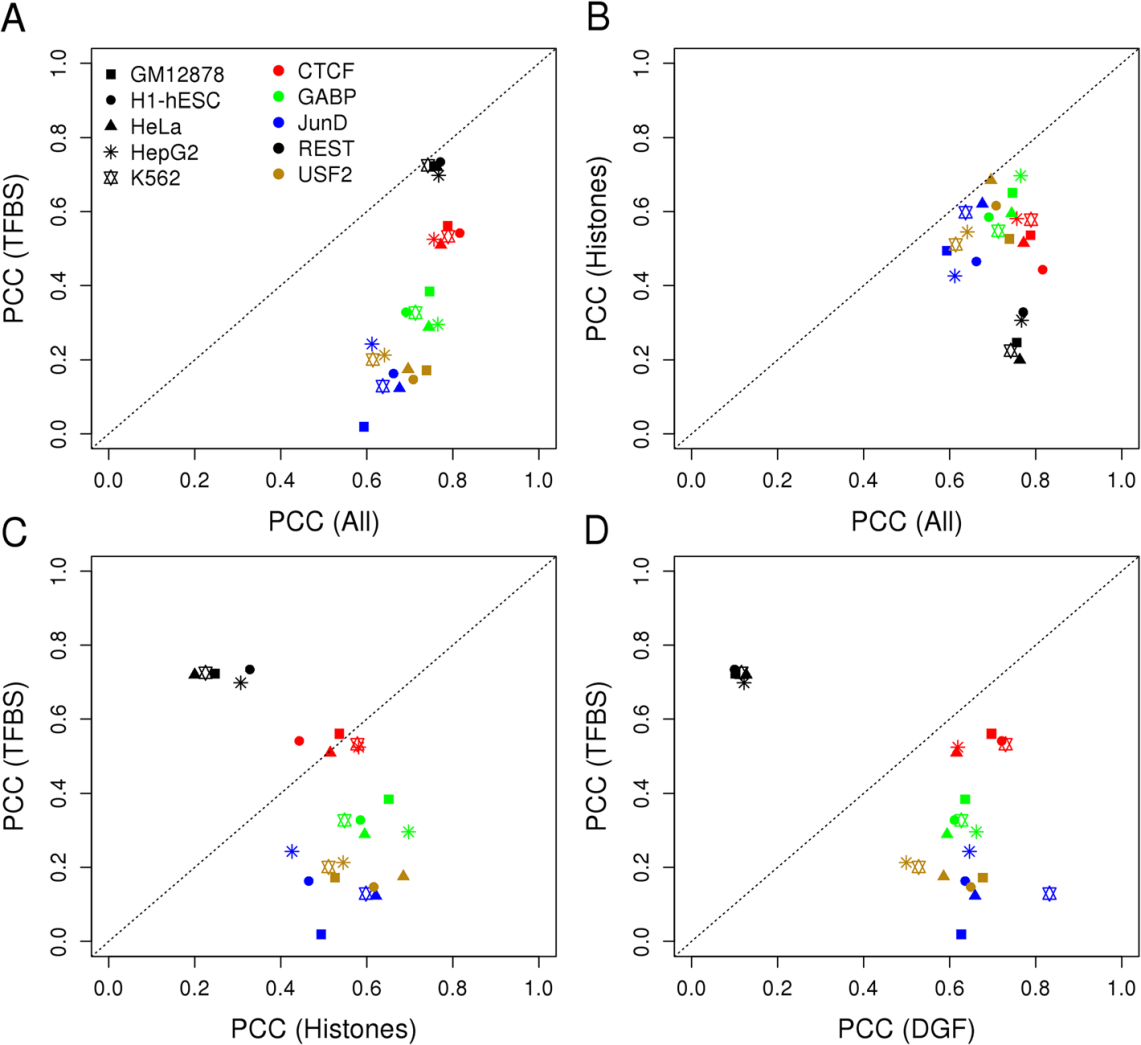
Regression of multiple factors in different cell lines. Each scatter plot compares the prediction accuracy of regression models (SVM) trained with two different feature sets. (**a**) TFBS vs all features, (**b**) histone marks vs all features, (**c**) TFBS vs histone marks and (**d**) TFBS vs DGF feature. “Histones” includes the following seven marks: H3K4me2, H3K4me3, H4K20me1, H3K9ac, H3K27ac, H3K27me3, H3K36me3

We then extended this type of regression-based machine modeling framework for predicting tag counts to three additional factors (REST, GABP and USF2) and three additional cell types (GM12878, HeLa and HepG2). The computational methods used were same as before, where we build models using 90 % of the data from each cell line/factor and prediction was carried out on the remaining 10 % test dataset. The purpose of this analysis was to obtain a more representative picture of the diversity of the TF-to-target-site recruitment processes operating in different cell types. We thus generated regression-based binding strength predictors with combinations of high performing features including histone marks, DGF, TFBS and PhastCons score using the predicted TFBS lists for each TF in all the cell lines. The cofactors Rad21 and FOSL1 were not used in this analysis to ensure fair conditions for all five TFs. The results are presented in a concise fashion in Fig. 6. Each subfigure compares two feature sets by means of a scatter plot. We first note that all factors (identified by different colors) form compact clouds in each plot, sometimes clearly separated from each other. The same is definitely not true for cell types (identified by different symbols). We conclude from this that the principles guiding the same transcription factors to its target sites are overall similar across cell types despite some undisputable cell-type specific effects for JunD, revealed by our results shown in Figs. 3 and 4.

On the other hand, the recruitment processes seem to be highly factor-specific. The most striking outlier among the five factors analyzed is REST whose binding strength is well predicted by its PWM score. However, all other features appear to be virtually useless for this factor. The fact that all dots lie on the main diagonal in Fig. 6a means that the prediction accuracy obtained with the PWM score cannot be increased by feeding more features to the learning algorithm even though histone modifications alone show a modest predictive value (Fig. 6b, R ~ 0.25). A dynamic interplay between REST binding and histone modifications was reported in a paper by Zheng et al. [28]. However, most of the histone marks selected in this study (except H3K27me3 and H3K36me3) did not show significant alterations with REST binding there. Perhaps a different selection of histone modifications would have shown better predictive ability of REST binding. This suggests that the histone modifications sometimes seen in the vicinity of a REST site are the consequence rather than the cause of REST binding to DNA. The results for REST are in sharp contrast to CTCF, where chromatin accessibility (DGF) and histone marks substantially increase prediction accuracy in all cell types. The other three factors can more or less be placed on a continuous line connecting CTCF to GABP, USF2 and JunD, in this order. The main discriminatory feature between these factors is the PWM score that varies in terms of its predictive value from high for CTFC to very low for JunD.

## Conclusions

We presented a quantitative framework to investigate and compare the role of sequence-intrinsic (tissue-invariant) and cell-type specific chromatin features in the biological processes that recruit TFs to their physiological target sites. Surprisingly distinct feature importance landscapes were observed for different TFs. The PWM score reflecting the intrinsic affinity of a TF to DNA was a good predictor of in vivo binding strength only for the two factors possessing an information-rich recognition motif (CTCF and REST). DGF came out high in terms of feature importance for all TFs studied except REST. Optimal performance was achieved with as few as three features suggesting high redundancy among the different features tested. DNA structural features, repressive and gene body associated histone marks were generally of little use in predicting in vivo binding site occupancy and strength. Our study highlights the immense value of large functional genomics data sets such as the ENCODE compendium for studying transcription regulation and illustrates the feasibility and effectiveness of pure *in silico* approaches in this field.

## Additional files

Additional file 1:
**Samples used in the study.** Details of samples used in the study. GEO and Jaspar ID are given. (PDF 84 kb) Additional file 2:
**Performance of machine learning algorithms.** Performance of various machine-learning algorithms to classify enriched and depleted CTCF sites (ENCODE peak list for K562 cell line) using multiple histone marks. (PDF 34 kb)
