# Supplementary material for: Predicting transcription factor site occupancy using DNA sequence intrinsic and cell-type specific chromatin features
Source: BMC Bioinformatics. 2016 Jan 11;17(Suppl 1):4. doi: 10.1186/s12859-015-0846-z (PMC4895346; doi:10.1186/s12859-015-0846-z)
Supplement: Additional file 1: — Samples used in the study. Details of samples used in the study. GEO and Jaspar ID are given. (PDF 84 kb) [file 12859_2015_846_MOESM1_ESM.pdf]

Additional File 1: Details of samples used in the study. GEO and Jaspar ID are given.

Numbers in parenthesis indicates tags shift value used for the sample.

	K562	***GM12878***	***HeLa-S3***	***HepG2***	***H1-hESC***
**ENCODE tags**					
CTCF	GSM733719 (112)	GSM733752 (95)	GSM733785 (113)	GSM733645 (84)	GSM733672 (76)
JunD	GSM935569 (58)	GSM935541 (40)	GSM935328 (73)	GSM935649 (69)	GSM935434 (62)
GABP	GSM803524 (72)	GSM803356 (49)	GSM803454 (52)	GSM803343 (47)	GSM803424 (63)
REST	GSM803440 (52)	GSM803349 (41)	GSM803478 (60)	GSM803344 (49)	GSM803365 (57)
USF2	GSM935356 (55)	GSM935558 (69)	GSM935561 (72)	GSM935646 (63)	GSM935380 (57)
**Histone marks (all factors)**					
H3K4me1	GSM733692				GSM733782
H3K4me2	GSM733651	GSM733769	GSM733734	GSM733693	GSM733670
H3K4me3	GSM733680	GSM733708	GSM733682	GSM733737	GSM733657
H4K20me1	GSM733675	GSM733642	GSM733689	GSM733694	GSM733687
H3K9ac	GSM733778	GSM733677	GSM733756	GSM733638	GSM733773
K3K27ac	GSM733656	GSM733771	GSM733684	GSM733743	GSM733718
H3K27me3	GSM733658	GSM733758	GSM733696	GSM733754	GSM733748
H3K36me3	GSM733714	GSM733679	GSM733711	GSM733685	GSM733725
**Other chromatin marks**					
DGF	GSM736629	GSM736496	GSM736564	GSM736637	GSM736582
Rad21	GSM803447 (46)				GSM803466 (66)
PolII	GSM803410 (60)				GSM803366 (72)
FOSL1	GSM803439 (63)				GSM803382 (55)
**TFBS (Jaspar ID)**	CTCF - MA0139.1	JunD -MA0492.1	GABPA - MA0062.2	REST - MA0138.2	USF2 - MA0526.1
