# Supplementary material for: Predicting transcription factor site occupancy using DNA sequence intrinsic and cell-type specific chromatin features
Source: BMC Bioinformatics. 2016 Jan 11;17(Suppl 1):4. doi: 10.1186/s12859-015-0846-z (PMC4895346; doi:10.1186/s12859-015-0846-z)
Supplement: Additional file 2: — Performance of machine learning algorithms. Performance of various machine-learning algorithms to classify enriched and depleted CTCF sites (ENCODE peak list for K562 cell line) using multiple histone marks. (PDF 34 kb) [file 12859_2015_846_MOESM2_ESM.pdf]

Additional File 2: Performance of various machine-learning algorithms to classify enriched and depleted CTCF sites (ENCODE peak list for K562 cell line) using multiple histone marks.

	SVM - RBF kernel	SVM – Polynomial kernel	Random forest (RF)
auROc	82.39	79.01	81.52
Time (s)	1024.89	6127.795	566.916
